# Therapeutic potential of biogenic and optimized silver nanoparticles using *Rubia cordifolia* L. leaf extract

**DOI:** 10.1038/s41598-022-12878-y

**Published:** 2022-05-25

**Authors:** Sandip Kumar Chandraker, Mishri Lal, Farheen Khanam, Preeti Dhruve, Rana P. Singh, Ravindra Shukla

**Affiliations:** 1 Laboratory of Bio-resource Technology, Department of Botany, Indira Gandhi National Tribal University, Amarkantak, Madhya Pradesh 484887 India; 2 Cancer Biology Laboratory, School of Life Sciences, Jawaharlal Nehru University, New Delhi, India

**Keywords:** Biological techniques, Biotechnology, Materials science, Nanoscience and technology

## Abstract

*Rubia cordifolia* L. is a widely used traditional medicine in the Indian sub-continent and Eastern Asia. In the present study, the aqueous leaf extract of the *R. Cordifolia* was used to fabricate silver nanoparticles (RC@AgNPs), following a green synthesis approach. Effect of temperature (60 °C), pH (8), as well the concentration of leaf extract (2 ml) and silver nitrate (2 mM) were optimized for the synthesis of stable RC@AgNPs. The phytofabrication of nanosilver was validated by UV–visible spectral analysis, which displayed a distinctive surface plasmon resonance peak at 432 nm. The effective functional molecules as capping and stabilizing agents, and responsible for the conversion of Ag^+^ to nanosilver (Ag^0^) were identified using the FTIR spectra. The spherical RC@AgNPs with an average size of ~ 20.98 nm, crystalline nature, and 61% elemental composition were revealed by TEM, SEM, XRD, and. EDX. Biogenic RC@AgNPs displayed a remarkable anticancer activity against B16F10 (melanoma) and A431 (carcinoma) cell lines with respective IC_50_ of 36.63 and 54.09 µg/mL, respectively. Besides, RC@AgNPs showed strong antifungal activity against aflatoxigenic *Aspergillus flavus*, DNA-binding properties, and DPPH and ABTS free radical inhibition. The presented research provides a potential therapeutic agent to be utilized in various biomedical applications.

## Introduction

Nanotechnology is an emerging paradigm of the twenty-first century, which deals with the manipulation of materials at the nanoscale and exploration of their versatile properties. Nanoparticles (NPs) have unique thermal, electric, chemical, magnetic, optical, and physical properties, unlike their bulk materials due to possessing quantum effects and high surface-to-volume ratio^1,2^. The use of NPs in biomedical science is well noticeable for several applications such as bio-imaging, cancer therapy, bio-detection of pathogens, drug and gene delivery, catalysis, biosensors, etc*.*^3,4^. Silver nanoparticles (AgNPs) are preferred over other metal-NPs in biomedical industries because of their tremendous antimicrobial applications^5–7^. AgNPs are used enormously in drug carriers, diagnostic technologies, coating of biomaterials and medical devices, tissue engineering and regeneration materials, challenging health-care approaches, and performance-enhanced therapeutic alternatives^8^. Although AgNPs have a promising future in nanoscience and biomedicine, significant efforts are needed to comprehend the intricate processes behind their biological interactions and pernicious effects^9^.

The common physical procedures for NPs’ synthesis, like attrition and pyrolysis, have various drawbacks, including imprecise surface formation, low fabrication rate, high manufacturing cost, and significant energy requirements. Other physicochemical methods (i.e. etching, chemical reduction, sol–gel technique, electro-explosion, laser ablation, etc.) include the use of toxic chemicals and the formation of poisonous substances^10^. The biological methods of NPs’ synthesis are gaining attention because of being non-hazardous, economical, eco-friendly, and devoid of the drawbacks of physicochemical processes. Phytosynthesis of NPs using leaves, stems, fruits, flowers, seeds, bark, and roots of the plants are highly cost-effective when compared to the microbial synthesis using bacteria, fungus, and algae. The tedious and expensive process of microbial culture and problems of contamination are the discouraging factors associated with microbe-mediated NP synthesis^11–13^. The plant-mediated synthesis of NPs is a simple, fast, reliable, economical, eco-friendly, and one-step method. The plant extracts possess various secondary metabolites viz. alkaloids, terpenoids, flavonoids, saponins, anthraquinones, tannins, etc., and are found to be responsible for the reduction of metal ion, and synthesis of NPs.

The global burden of cancer is rising with 18.1 million active cases and 9.6 million cancer deaths as reported in GLOBOCAN 2018^14^. Certain AgNPs have been shown to possess anticancer, anti-inflammatory, anti-oxidant, and antimicrobial activities^15–17^.

*Rubia cordifolia* L., commonly known as ‘Indian madder’ or ‘Manjishta’ is a perennial climbing herb, belonging to the family: Rubiaceae^18^. The root of the plant is widely used in traditional medicine in India, Japan, Korea, and China for treating tuberculosis, wounds, menoxenia, and rheumatism. *R. Cordifolia* has some anthraquinones and anthraquinone derivatives to support various therapeutic properties, including anti-fungal, anti-oxidant, anti-inflammatory, anti-bacterial, anti-tumor, and anti-cancer activities^18–21^. Copious literature is available on the uses of the root, whereas, leaves are usually discarded for no use. No study has been undertaken to explore the therapeutic properties of leaves.

The study aimed to synthesize and characterize biogenic AgNPs from aqueous leaf extract of *R. Cordifolia*, as well as to explore their anticancer, DNA-binding, antifungal, and antioxidant properties.

## Material and methods

### Plant materials

*R. cordifolia* leaves were collected in January 2019 from the forest area of Amarkantak, Madhya Pradesh, India. The identification of *R. cordifolia* was certified by a subject expert, and a voucher specimen (DOB/07/RC/120/2019) was deposited in the Botany department, IGNTU, Amarkantak, MP, India. The collection of the plant material and related studies complies with relevant institutional, national, and international guidelines and legislation.

### Chemicals

All the chemicals [Silver nitrate (AgNO_3_), CT-DNA, Tris buffer, potato dextrose broth (PD-B), potato dextrose agar (PDA), 2,2-Diphenyl-1-picrylhydrazyl (DPPH) and 2,2-Azino-bis-3-ethylbenzothiazoline-6-sulphonic acid (ABTS)] used were AR grade and purchased from Himedia (Mumbai, India). Double distilled water (DDW) was used throughout the experiments.

### Preparation of leaf extract

**Figure 1:**
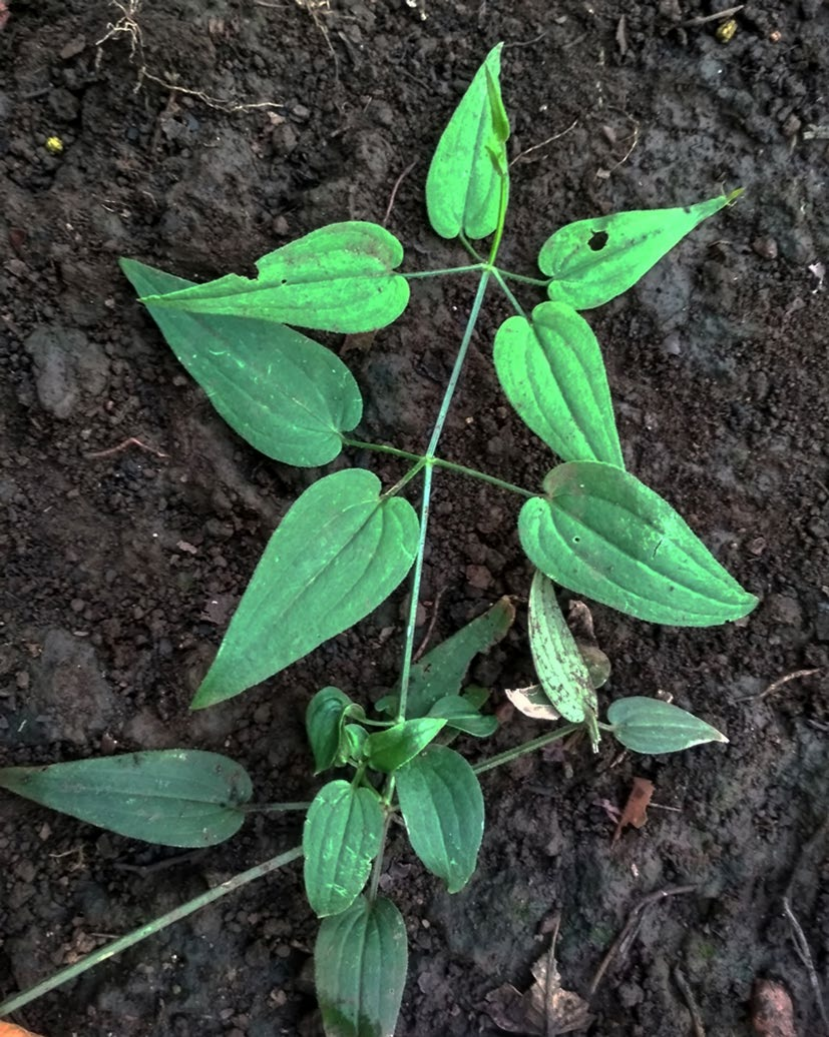
Plant of *R. cordifolia* L.

Approximately, 10 g of leaves of *R. cordifolia* (Fig. 1) were weighed through the analytical balance, washed twice with DDW, and dried at room temperature. The chopped leaves were placed in a 250 mL conical flask with 100 mL of DDW, boiled for half an hour at 70 ^0^C, and filtered twice using Whatman No. 1 filter paper. The filtrate was kept at 4^0^C for further use.

### Phytochemical analysis

The principal phytochemicals in *R. cordifolia* leaf extract (RCLE) were examined following the procedure of Chandraker et al.^22^. Furthermore, for the detection of free and combined anthraquinones, Borntrager’s test was adopted following Ukwubile et al.^23^.

### Optimized synthesis of AgNPs

Several factors, including temperature, pH, AgNO_3_ concentration, and RCLE, were optimized to synthesize saturated and stable *R. cordifolia*-mediated RC@AgNPs. To optimize the suitable temperature, four reaction mixtures of 1 mL of RCLE and 9 mL of AgNO_3_ (1.0 mM) were mixed and incubated at four different temperatures, viz., 20, 40, 60, and 80 °C for 60 min. To optimize the pH, the above experimental process was repeated at different pHs (2, 4, 6, 7, and 8). Four different volumes of RCLE (9.5, 9, 8.5, and 8 mL) were treated with corresponding volumes (0.5, 1.0, 1.5, and 2.0 mL) of 1 mM AgNO_3_ solution to find the optimum concentration of RCLE. Similarly, to optimize AgNO_3_ concentration, 0.2, 0.5, 1.0, and 2.0 mM of 1 mL AgNO_3_ were treated individually with 9 mL of RCLE. All the four experimental set-ups were incubated for 60 min and analyzed thereafter with UV–visible absorption spectroscopy.

### Characterization of RC@AgNPs

Phytosynthesis of RC@AgNPs was confirmed by UV–visible spectroscopy (Shimadzu UV-1800). To investigate the nanosize and structure of RC@AgNPs, transmission electron microscopic (TEM-Technai G20, FEI) and X-Ray Diffraction (XRD) [Bruker-D8 at 30 kV and 20 mA current with Cu K (I = 1.54 A)] studies were performed. The topology and elemental composition of RC@AgNPs were determined by Energy Dispersive X-ray linked Scanning Electron Microscopy (SEM) (EVO 18; Carl Zeiss, Germany). The average size (size distribution), dispersity, and stability (zeta potential) of RC@AgNPs in the aqueous medium were calculated by Anton Paar (Litesizer 500). Fourier Transform Infra-Red (FTIR) spectra of RCLE and RC@AgNPs were obtained from Thermo Scientific Nicolet 5.

### Applications of RC@AgNPs

#### Anticancer activity

The culture and maintenance of cells were done in Dulbecco's Modified Eagle’s Medium (DMEM) with 10% Fetal Bovine Serum (FBS) and 1% antibiotic and antimitotic solution at 37 °C with 5% CO_2_. For the experiment, NPs were suspended in dimethylsulfoxide (DMSO) with the concentration of DMSO in the medium not exceeding 0.1% in all the treatment groups. The standard 3-(4,5-dimethylthiazol-2-yl)-2,5-diphenyl tetrazolium bromide (MTT) assay was used to assess the anticancer efficacy of RC@AgNPs against A431 (squamous cell carcinoma cell line) and B16F10 (melanoma cell line). Both the cell types were seeded at the concentration of 5000 cells/well in 96 well plates. After incubating the cells for 24 h, treatments were given to cells for 24 and 48 h with different concentrations (10, 25, 50, and 100 µg/mL) of RC@AgNPs. MTT was added to the wells, and the plate was further incubated for 4 h. The added MTT was replaced gently by 100 µl of DMSO and incubated briefly for ten minutes at 37 ^0^C, with the absorption measured in a microplate reader at 570 nm. The percentage of cell viability was estimated using the following formula:

$$\% Cell\, viability = \left[\frac{Average\, absorbance\, of\, the\, treated\, sample }{Average\, absorbance\, of\, the\, control\, sample}\right]\times 100$$

#### DNA interaction activity

Calf-thymus DNA (CT-DNA) solution was prepared with Tris buffer (pH = 7.2). Different concentrations of RC@AgNPs (0.010–0.090 nM) were treated with a concentration of CT-DNA (250 µM). The interaction between RC@AgNPs and CT-DNA has been carefully monitored in a UV–visible spectrophotometer by absorption-titration experiments.

#### Antifungal activity

For antifungal activity, the RC@AgNPs were evaluated against a food-spoiling, saprophytic, pathogenic, aflatoxigenic, and ubiquitous fungi *Aspergillus flavus*. The fungal strain (No. 277) was procured from the Microbial Type Culture Collection and the Gene Bank (MTCC) of the Institute of Microbial Technology (IMTECH), Chandigarh, India. Contact assay was adopted to determine antifungal activity, following Shukla et al.^24^. Five different doses of RC@AgNPs (1.25, 2.5, 5.0, 10.0, and 20.0 mg) were taken in separate Petri dishes (90 mm) and 20 mL PDA was poured into each petri dish. Finally, 5 mm fungal discs taken from the *A. flavus* colony (One week old) were inoculated at the center of RC@AgNPs and PDA-containing plates. The inoculated Petri dishes were incubated for seven days in the dark at 27 °C to determine the inhibition of the mycelial growth of the fungi. The negative control plates without RC@AgNPs, and positive control plates with RCLE and 1 mM silver nitrate solution, separately were also incubated under the same growth conditions. The effect of RC@AgNPs, RCLE and 1 mM silver nitrate on the growth of *A. flavus* was tested in triplicates. After seven days, the radial colony's growth and photographic record were established.

The percentage of fungal inhibition was calculated based on the rate of radial growth inhibition, as follows:

$${\varvec{I}}{\varvec{R}}{\varvec{G}}\boldsymbol{ }\left(\boldsymbol{\%}\right)=\left[\frac{{{\varvec{R}}}_{1}-{{\varvec{R}}}_{2}}{{{\varvec{R}}}_{1}}\right]\times 100$$

where IRG = Inhibition of the radial growth, R1 = Control's radial growth, and R2 = Radial growth in treatments, IRG was estimated using the mean ± standard error (SE) of the triplicate data.

#### Antioxidant capability

The antioxidant properties of RC@AgNPs were determined using the DPPH and ABTS free radical scavenging tests described by Chandraker et al.^25^. Using UV–visible spectroscopy, the antioxidant potential of RC@AgNPs was tested in the presence of DPPH and ABTS free radicals, and ascorbic acid was used as a standard reference. The percentage of scavenging activity was calculated using the following formula.

$$\% scavenging\, activity = \left[\frac{Absorbance\, of\, control-Absorbance\, of\, sample }{Absorbance\, of\, control}\right]\times 100$$

### Statistical analysis

OriginPro 8.5 and ImageJ software were used throughout the experiments, and all tests were performed in triplicate, with data presented in mean ± SE. Data for the MTT assays were examined using the Student's t-test, with P < 0.05 considered statistically significant.

## Results and discussion

### Phytochemical analysis

Phytochemical analysis of RCLE revealed the presence of flavonoids, tannins, phlobatannins, saponins, and anthraquinones (Table 1). Our findings are almost similar to that of previous reports where Chandrashekar et al. studied the phytoconstituents of *R. cordifolia* root^26^. These phytochemicals may act as reducing, capping, and stabilizing agents during the synthesis of AgNPs from RCLE. Phytochemicals are supposed to reduce Ag^+^ to Ag^0^ and synthesize RC@AgNPs. Some previous studies confirmed that tannins^27^, and saponins^28^ are responsible for reducing the metal ions to metallic-NPs. Anthraquinones are reported to be present in all the parts of *R. cordifolia*^26^. Purpurin (1,2,4-trihydroxyanthraquinone) and munjistin (1,3,-dihydroxy-9,10-dioxo-9,10-dihydroanthracene-2-carboxylic acid) are two predominant anthraquinones present in *R. cordifolia*, and supposed to be responsible for its pharmacological properties^29–31^.

**Table 1:** Phytochemical analysis of RCLE.

S. no	Phytochemicals	Test	Result
1	Alkaloids	Mayer’s test, Wagner test Dragendroff test	−ve −ve
2	Saponins	Foam test	+ ve
3	Anthraquinones	Borntrager’s test	+ ve
4	Tannins	Ferric chloride test	+ ve
5	Flavanoids	Ferric chloride test and Lead acetate test	+ ve
6	Phlobatannins	HCL test	+ ve
7	Diterpenes	Copper acetate test	−ve
8	Steroids	Salkowski test	−ve
9	Triterpenes	Salkowski test, Liberman test	−ve

+ Present and −Absent.

### Biogenic synthesis of RC@AgNPs

**Figure 2:**
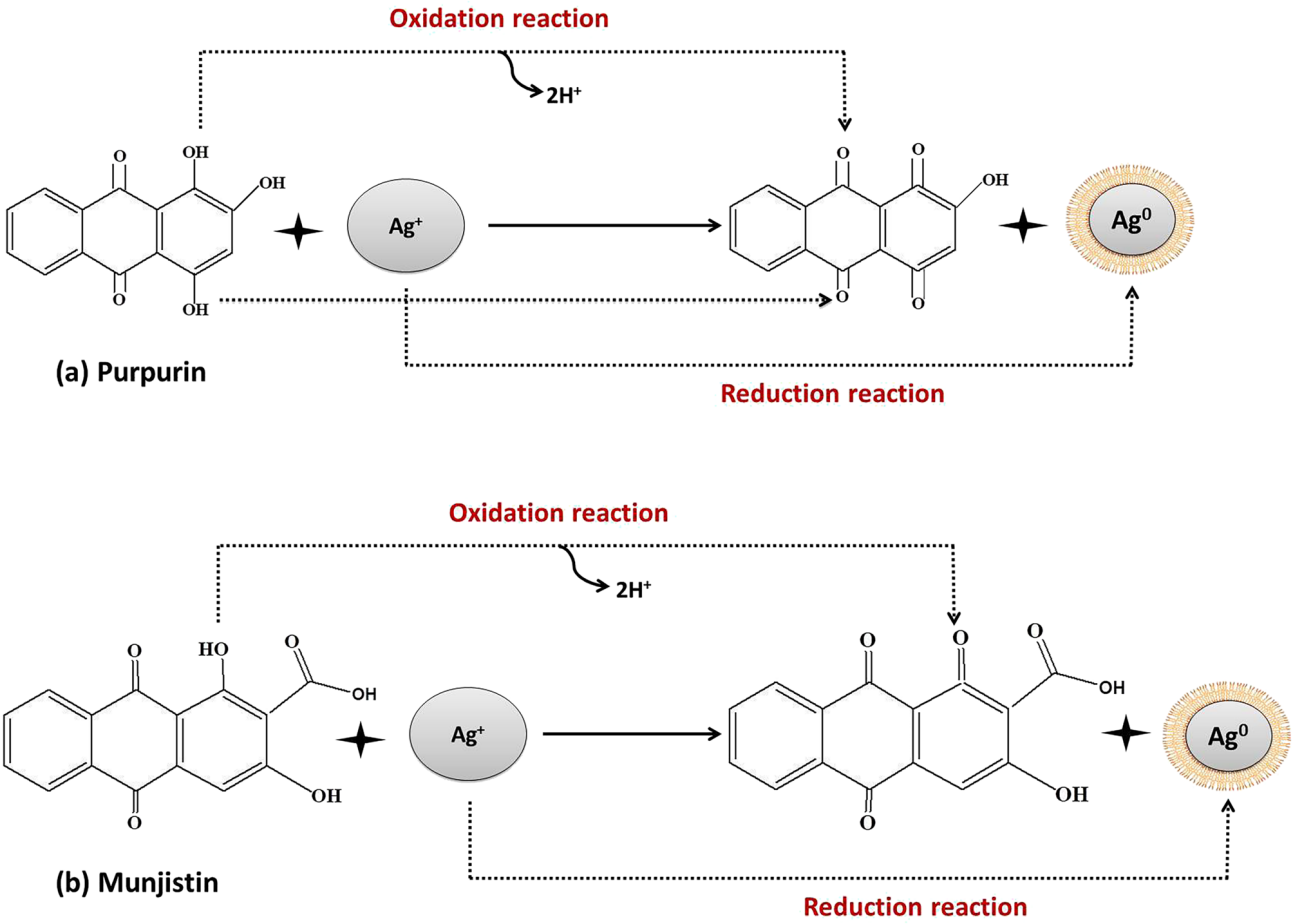
A possible mechanism showing the role of two anthraquinones in the reduction of Ag^+^ to nanosilver (Ag^0^).

**Figure 3:**
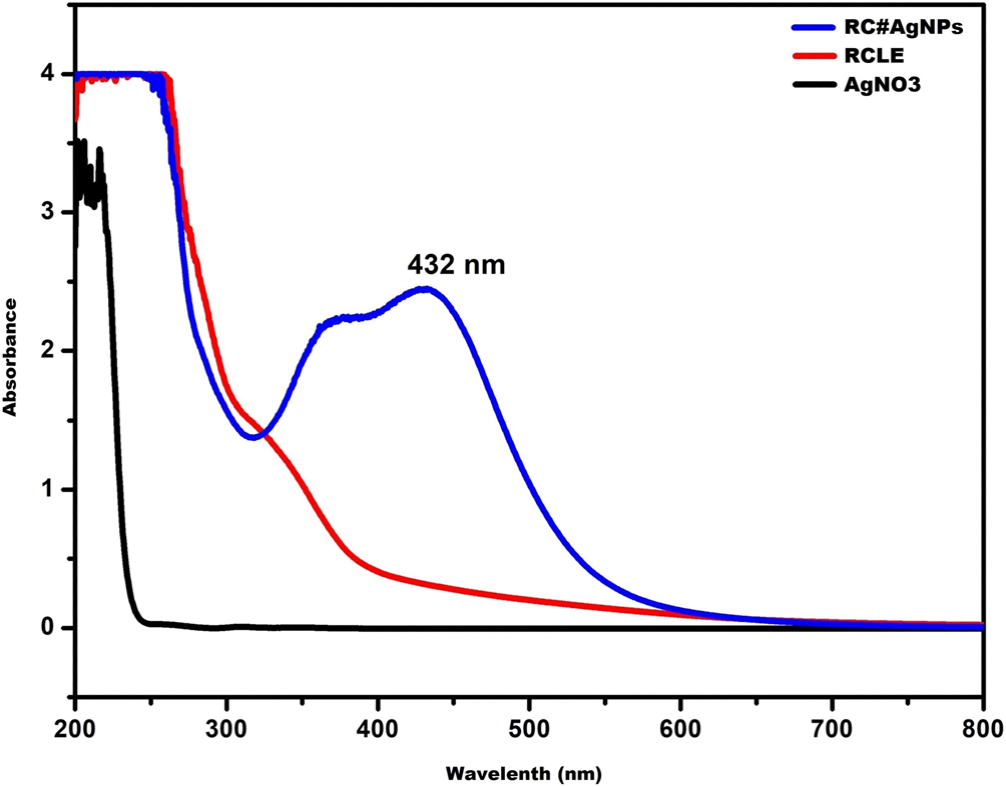
UV–visible analysis of RC@AgNPs, RCLE, and silver nitrate.

Silver nanoparticles were synthesized following an eco-friendly method^32^. RCLE was used as a capping, stabilizing, and a reducing agent to fabricate RC@AgNPs from AgNO_3_. The schematic illustration of nanosilver synthesis from two major anthraquinones of RCLE is shown in Fig. 2. The change of color from light-green to reddish-brown has been considered as RC@AgNPs synthesis. Changes in the color of the reaction mixture are due to the conversion of Ag^+^ to Ag^0^, and they were easily monitored by Ultraviolet–visible spectroscopy. Figure 3 represents the UV–visible analysis of RC@AgNPs, RCLE, and silver nitrate solution. In Fig. 3 it is clearly shown that RC@AgNPs have a signature AgNPs’ absorbance peak at 432 nm, whereas, RCLE and AgNO_3_ do not. Another peak shown around 380 nm may originate from an interband transition^33^. RC@AgNPs displayed a negligible shift in λ_max_ from 432 to 435 nm, after 5 months of synthesis, thus found to be stable.

Due to surface plasmon resonance (SPR), AgNPs tend to exhibit a characteristic absorption maximum (λ_max_) in the 400–500 nm range^34^. The absorption maximum of *Justicia adhatoda* mediated AgNPs was 463 nm^35^, *Sonchus arvensis* mediated AgNPs was 440 nm^36^, *Equisetum arvense* mediated AgNPs was 488 nm^37^, and *Withania coagulans* mediated AgNPs were 445 nm^38^, SPR is a cumulative oscillation of conduction electrons triggered by incoming light at the interface between (-)ive and ( +)ive materials. The condition is regulated by the capping agents on Ag-surface. Plants have variations in their phytochemical constituents, and thus, varied functional groups act as capping, reducing, and stabilizing agents. Therefore, different plant-mediated AgNPs show variable λ_max_, size, and stability.

### Optimization of biogenic RC@AgNPs

**Figure 4:**
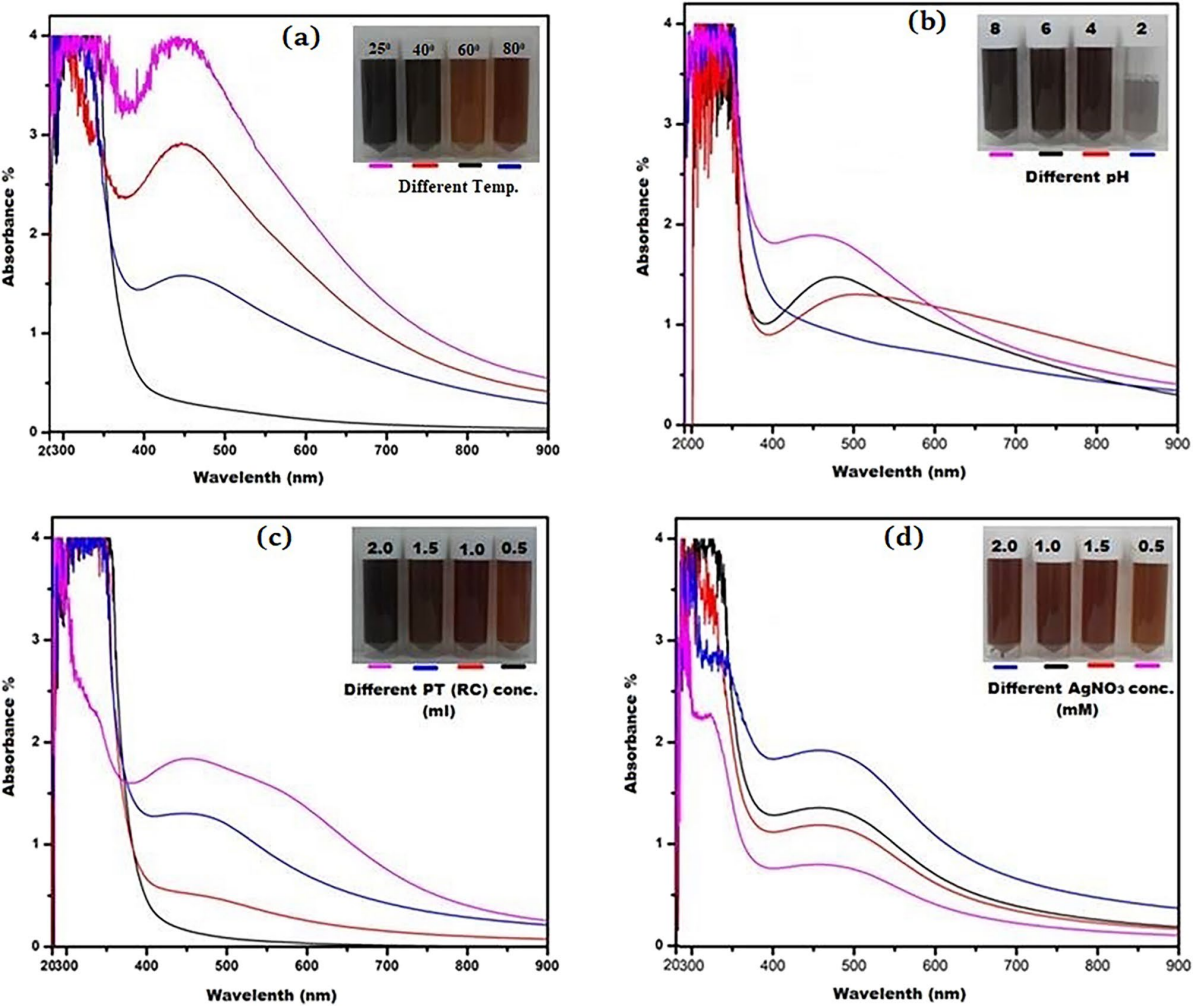
(**a**) UV–vis spectra of RC@AgNPs: at different temperatures of 25, 40, 60, and 80 ºC; (**b**) at different pH of 2, 4, 6, and 8; (**c**) at different amount of RCLE i.e. 0.5, 1.0, 1.5 and 2 ml; (**d**) at different AgNO_3_ concentrations of 0.5, 1.0, 1.5, and 2.0 mM. (Except Fig. 4c, the remaining experiments had the same concentration of RCLE; Similarly, except Fig. 4d, the concentration of AgNO_3_ was the same in the rest of the experiments).

The yield of NPs is influenced by the reaction conditions of the biogenic synthesis process. Various reaction parameters explicitly alter the size distribution and reaction rate of the NPs synthesis. Figure 4 shows the alterations in the color of the reaction mixture under several experimental conditions^39^.

The reaction temperature was indeed a vital aspect that expressively related to the rate of biogenic synthesis of RC@AgNP. To investigate the effect of temperature on the phytosynthesis of RC@AgNPs, 1 mL of RCLE was treated with 9 mL of 2 mM AgNO_3_ in 4 different vials and kept at temperatures ranging from 20 °C to 80 °C. According to the findings, increasing the temperature in the reaction mixture increased the biogenic synthesis of RC@AgNPs (Fig. 4a). The considerable RC@AgNP synthesis was observed at 60 °C, even though absolute RC@AgNP synthesis was reported at 80 °C. It shows that the entire synthesis of RC@AgNPs may be achieved at temperatures of 60 °C and 80 °C, where RCLE phytochemicals conduct optimal reduction and stabilisation^40^. The absorbance peak at 80 °C seems distorted due to the accumulation of RC@AgNPs generated by high-temperature biogenic production.

The pH value is always crucial for any reaction. In the biogenic synthesis of AgNPs, pH is essential. The color of the reaction medium, the strength of the SPR peak, and the shape and size of the NPs were investigated to be pH-dependent. RC@AgNPs absorbance peak rises with increasing pH, and the maximum fabrication of RC@AgNPs occurred at pH 8. In our investigation, the acidic media with pH 2 and 4 had a lower absorption peak than pH 6 and 8. As a result, we concluded that an alkaline pH of 8 is ideal for the synthesis of RC@AgNPs (Fig. 4b). According to our observations, absorption increases with rising pH, indicating that an alkaline condition is preferable to an acidic one for the synthesis of NPs. During the synthesis of NPs, the change in the pH of the reaction solution affects the ability of the phytochemicals to reduce silver ions. Vanaja et al. also proposed the increase in absorption when pH rises, and it can sometimes affect the form, size, and production of NPs^41^. Qian et al. and Handayani et al*.* found similar results with *Pometia pinnata* (Matoa) leaf extract and *Epicoccum nigrum* (fungus) mediated green synthesis of AgNPs, that an alkaline medium is more pertinent than an acidic medium^42,43^. Sintubin et al. suggested that higher pH enhances the race between H^+^ and Ag^+^ for bond formation with phytochemicals; thus, the higher pH gave better results than the lower pH for the synthesis of AgNPs^44^.

The RCLE is another key component in the biosynthesis of RC@AgNPs. The absorption spectra of RC@AgNPs under visible range were established using various RCLE concentrations (0.5, 1, 1.5, and 2 mL), where the AgNO_3_ concentrations were held at 2 mM constant. Figure 4c indicates no absorption spectrum at 0.5 RCLE concentrations. On increasing the concentration of RCLE, absorption peaks were observed, and at 2.0 mL RCLE concentration, maximum absorbance was found. The increasing concentration of biomolecules involved in metal reduction has enhanced the synthesis of environmental-friendly NPs. Similar results were also found with the *Pongamia pinnata* leaf extract^45^ and *Citrullus lanatus* fruit rind extracts^46^.

The concentration of AgNO_3_ also affects the phytosynthesis of AgNPs, significantly. Multiple concentrations (0.2, 0.5, 1, and 2 mM) of AgNO_3_ solution were used to determine their effect on RC@AgNPs synthesis. AgNO_3_ concentration of 2.0 mM results in maximum RC@AgNPs synthesis, with an absorbance peak at 432 nm (Fig. 4d). Poor peaks at lower concentrations of 0.2 and 0.5, and 1.0 mM AgNO_3_ could be due to the deficient availability of Ag^+^ ions in the reaction mixture.

### Characterization of RC@AgNPs

#### XRD analysis

**Figure 5:**
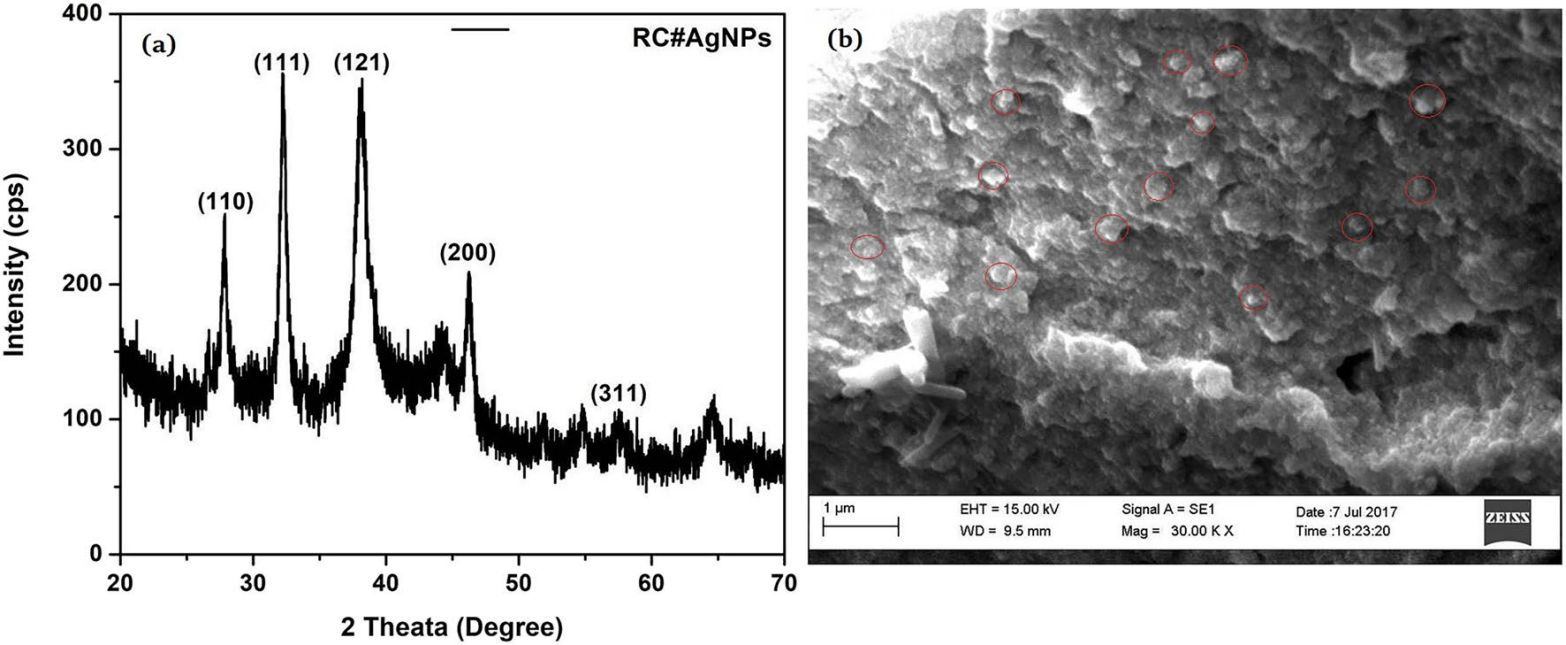
(**a**) XRD spectra, and (**b**) SEM images of biogenic synthesized RC@AgNPs.

The crystalline nature of the particles was confirmed with XRD. Figure 5a shows an XRD pattern of the AgNPs synthesized by using RCLE. The XRD pattern showed many Bragg reflections based on Ag's face-centered cubic (fcc) structure. The nanosize of RC@AgNPs was confirmed after matching the obtained XRD spectrum with the standard (JCPDS file no. 84–0713). The peaks at 2 theta values of 27.85°, 32.19°, 38.19°, 46.24°, and 57.52°, demonstrate the (110), (111), (121), (200), and (311) Bragg's reflections, which may be indexed based on the fcc structure of metallic silver^47^. XRD results thus confirmed the crystalline nature of RC@AgNPs.

#### SEM and EDX analysis

**Figure 6:**
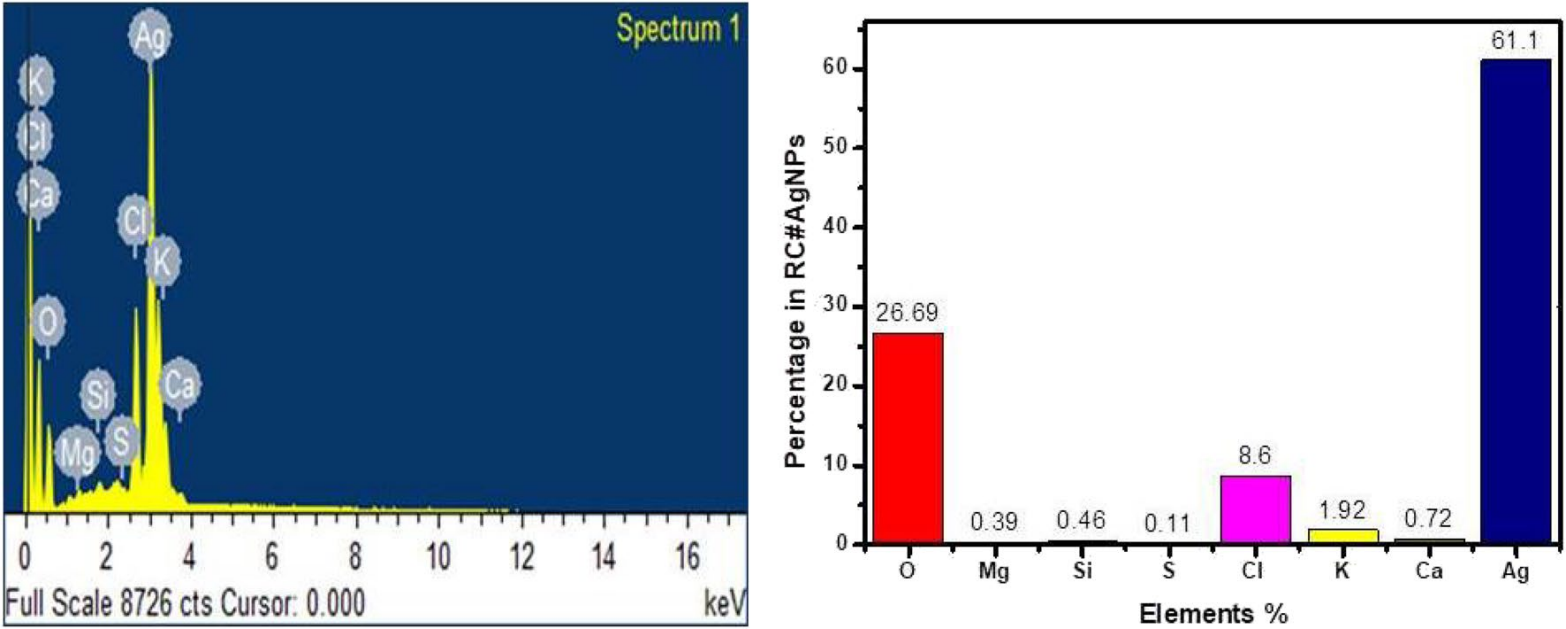
(**a**) EDX spectrum of RC@AgNPs synthesized from RCLE, and (**b**) Quantitative estimation of EDX data of RC@AgNPs.

SEM image (Fig. 5b) shows the granular appearance of RC@AgNPs. Different elements' composition in RC@AgNPs is confirmed by EDX analysis at 3 keV. In Fig. 6a the EDX spectrum reveals a high elemental peak, showing metallic silver. According to the quantitative assessment, metallic silver has a more significant weight, 61.1%, while O, Mg, Si, S, Cl, K, and Ca have 26.69, 0.39, 0.46, 0.11, 8.6, 1.92, and 0.72%, respectively (Fig. 6b).

#### TEM analysis

**Figure 7:**
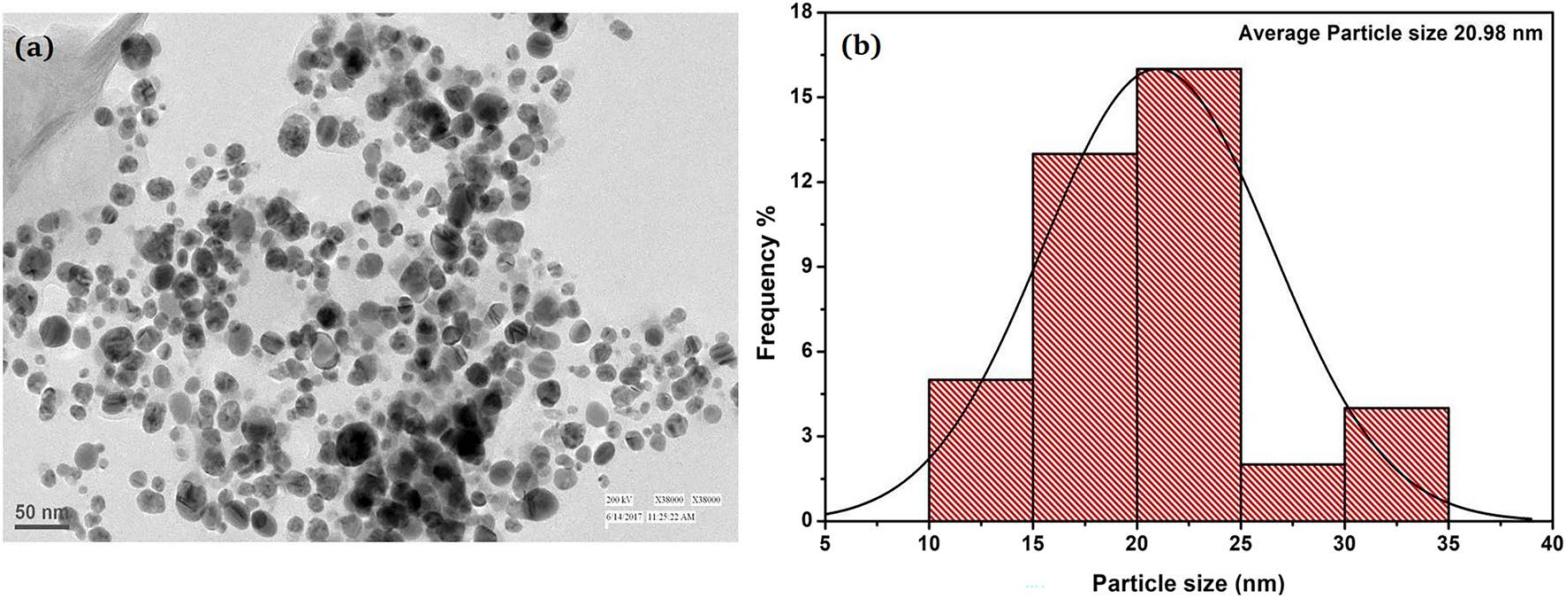
(**a**) TEM images of RC@AgNPs, and (**b**) Particle size distribution histogram of RC@AgNPs from the TEM images.

Figure 7a shows TEM images of RC@AgNPs that illustrate the formation of isotropic spherical AgNPs. The particle size distribution histogram of RC@AgNPs using TEM images revealed an average particle size of 20.98 nm (Fig. 7b). This particle size spectrum was obtained using spectroscopy-based SPR and XRD.

#### FT-IR spectral analysis

**Figure 8:**
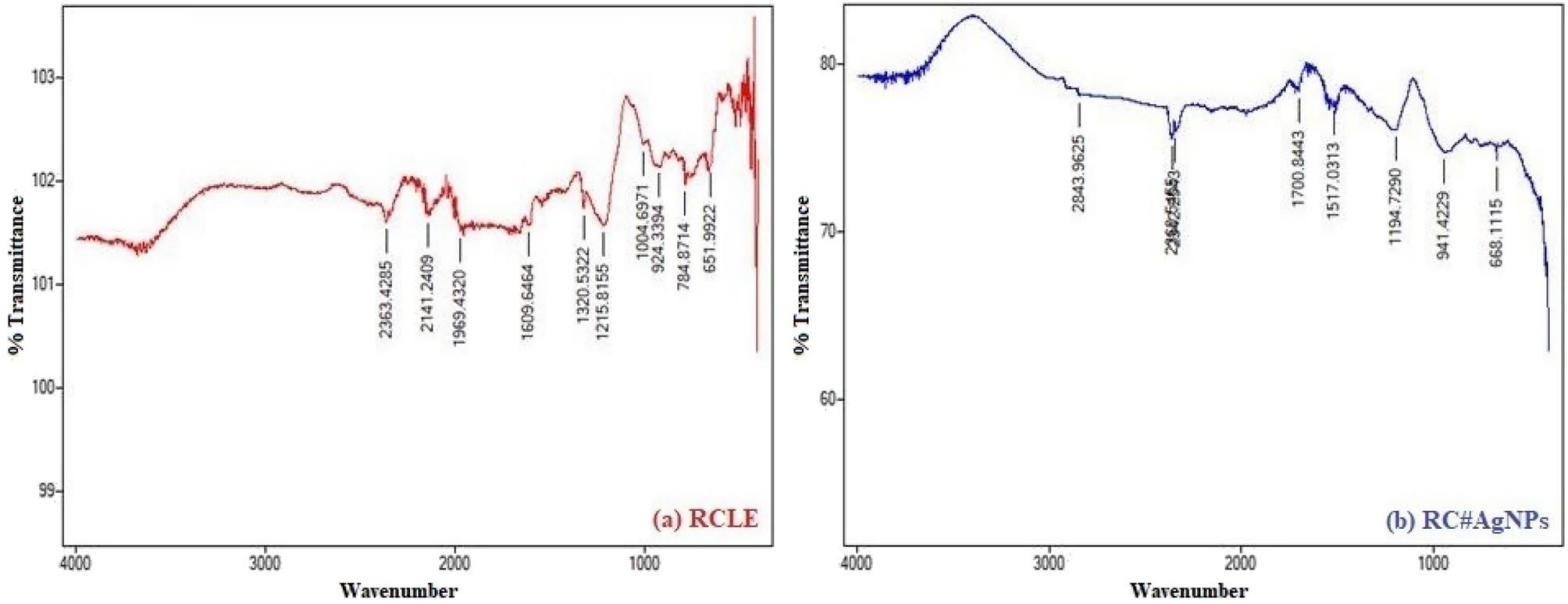
FTIR spectra of biogenic synthesized, (**a**) RC@AgNPs, and (**b**) RCLE.

FT-IR analysis of RCLE and RC@AgNPs was done to reveal functional groups present in the extract of *R. cordifolia* and present on the surface of AgNPs. Table 2 shows the wavenumber and interpretation of possible functional groups of RC@AgNPs and RCLE. Figure 8a–b shows the FT-IR spectrum of RC@AgNPs and RCLE. The leaf extract contains various phytochemicals, which might be responsible for the reduction and stabilization of Ag^+^ to Ag^0^ and form RC@AgNPs. A similar pattern of the result was found with *Ageratum houstonianum* extracts and resulting NPs^22^.

**Table 2:** FT-IR analysis of RCLE and RC@AgNPs; it’s probable functional groups.

RCLE wavenumbers	Probable function groups	RC@AgNPs wavenumbers	Probable function groups
651.9922	Aromatic-H bending	668.1115	Aromatic-H bending
784.8714	N–H bend		
924.3394	C=C–H bending	941.4229	C=C–H bending
1004.6971	O–C stretching	1194.7290	C–O stretch
1215.8155	O–C stretch	1517.0313	N–H bend
1320.5322	O–H bend	1700.8443	C=O stretching
1609.6464	N–H bend	2342.2543	C–H stretching
1969.4320	Polystyrene	2843.9625	N^+^–H stretch stretch
2141.2409	–N–C=O stretch

#### Zeta particle size and zeta potential of RC@AgNPs

**Figure 9:**
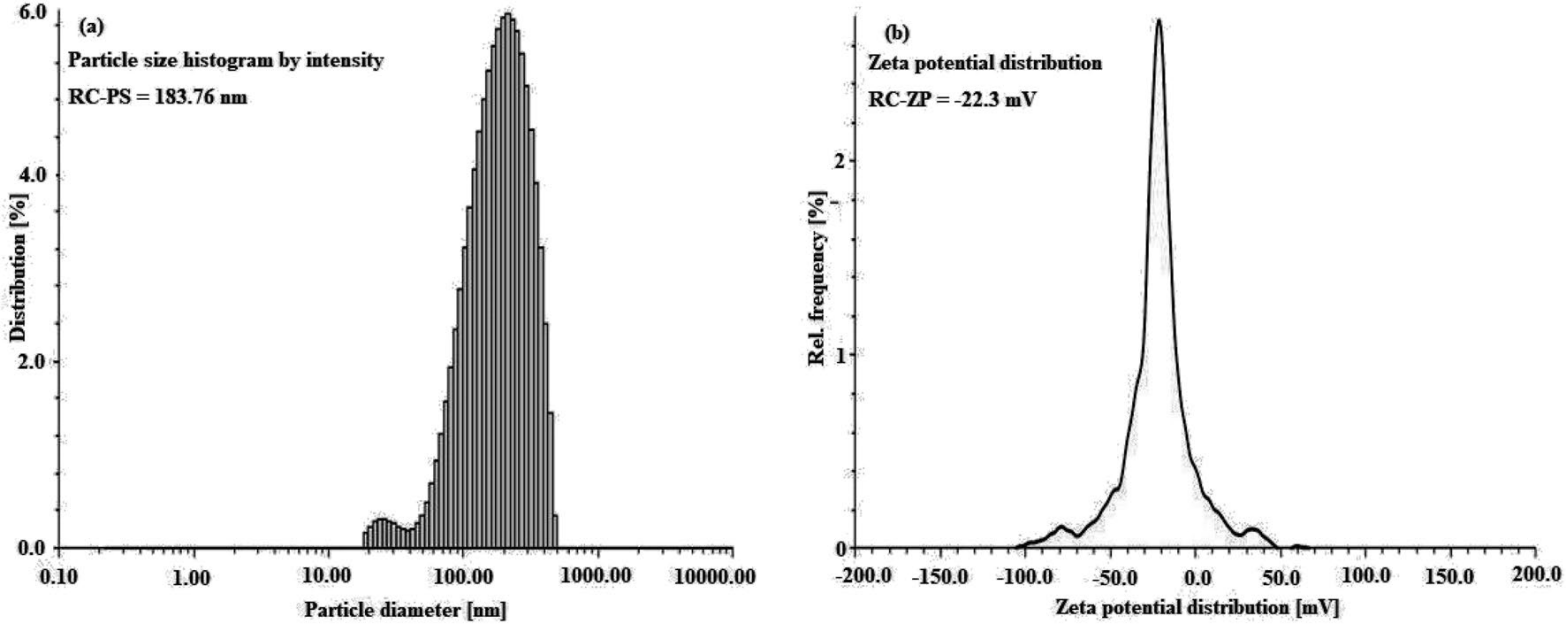
(**a**) Zeta particle size, and (**b**) Zeta potential of RC@AgNPs.

Dynamic light scattering (DLS) and Laser Doppler Electrophoresis (LDE) measurements were carried out to determine the particle size distribution and zeta potential of RC@AgNPs, respectively in an aqueous solution. The resulting average zeta particle size is 183.76 nm, with a polydispersity index (PDI) of 26.2, as shown in Fig. 9a. Because DLS measurements were dependent on the hydrodynamic radius of the NPs in the aqueous environment, the grain size distribution was found to be greater than the normal NPs reported by TEM and XRD studies. The related zeta potential of RC@AgNPs was found to be −22.3 mV in Fig. 9b, indicating that RC@AgNPs are remarkably stable. Kokila et al. found similar DLS and zeta potential results with *Carica papaya* peel extract mediated biosynthesized AgNPs^48^.

## Applications of RC@AgNPs

### Anticancer activity

**Figure 10:**
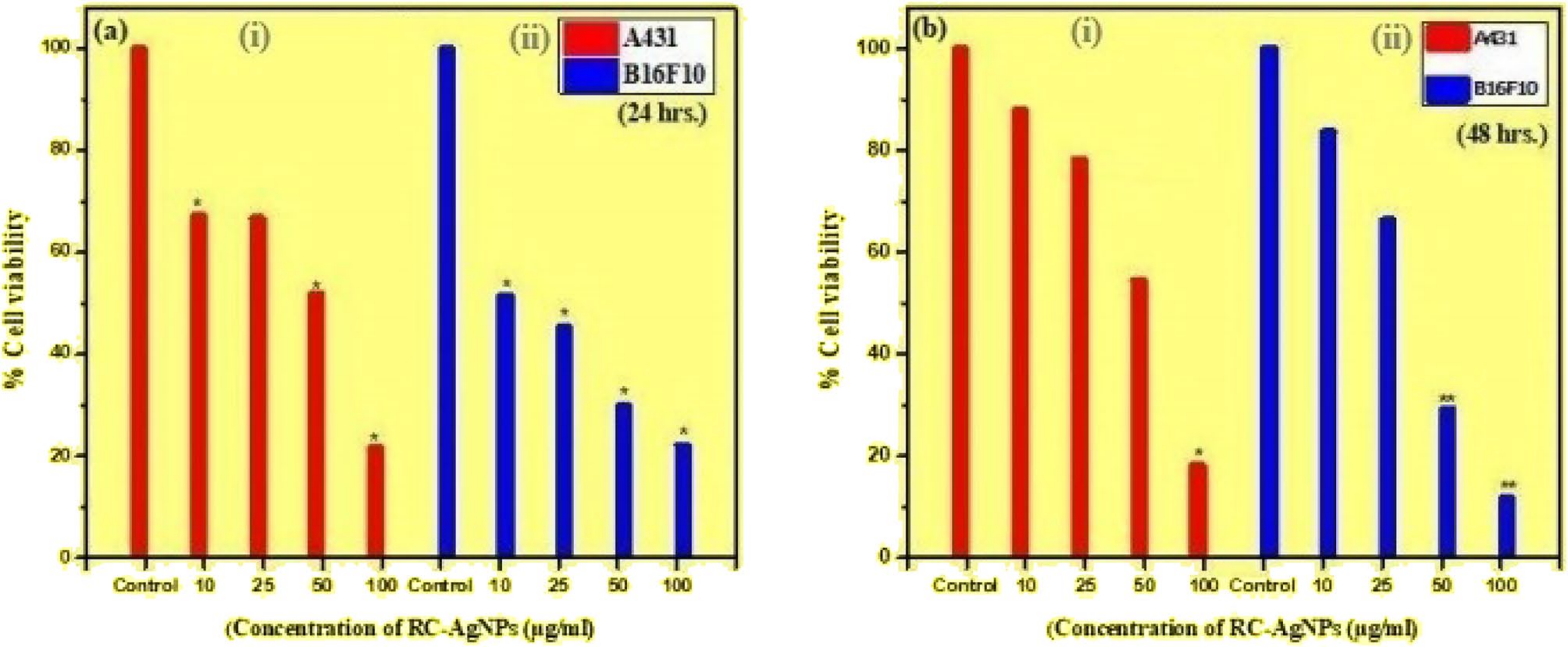
(**a**) A431 (cell carcinoma squamous line) and (**b**) B16F10 (cell melanoma line) viability percentages after incubation at different times (24 and 48 h) with varying concentrations of biogenic RC@AgNPs from RCLE.

MTT assay was performed on A431 and B16F10 cells to determine the cytotoxic activity of RC@AgNPs. In this assay, yellow color dye 3-(4,5-dimethylthiazol-2-yl)-2,5-diphenyl tetrazolium bromide (MTT) is reduced by the mitochondrial enzyme succinate dehydrogenase, which leads to the formation of purple-blue formazan crystals. Since it is a colorimetric measurement, the absorbance is recorded for the results. Higher the cytotoxicity of NPs lower will be the viability of cancer cells (human squamous cell carcinoma: A431 and mouse melanoma: B16F10). The results of the in vitro assays suggest the cytotoxic activity of RC@AgNPs against the A431 and B16F10 cells (Fig. 10). In the results, it was found that there was a concentration-dependent decrease in the cell viability of both A431 and B16F10 cells at both time points. In the case of A431 cells, after 24 h of treatment, there was a 10%, 20%, 50%, and 80% decrease in cell viability at 10, 20, 50, and 100 µg/mL of concentration, respectively (Fig. 10a,i). After 48 h, the decrease was around 50 and 80% at higher concentrations of 50 and 100 µg/mL (Fig. 10b,i). In B16F10 cells after 24 h of treatment the decrease in cell viability was 20%, 30%, 70% and 90% at 10, 20, 50 and 100 µg/mL of concentration respectively (Fig. 10a,ii). Treatment with RC@AgNPs for 48 h caused a 50% decrease at a lower concentration of 10 µg/mL and a higher concentration of 100 µg/mL, causing a remarkable ~ 80% decrease in the cell viability of B16F10 cells (Fig. 10b,ii). The IC_50_ value of RC@AgNPs against A431 and B16F10 cells were 54.09 and 36.63 µg/mL, respectively. There are some reports on the cytotoxic activity of AgNPs against different cancer cell lines. Mainly, the activity is attributed to the oxidative stress induced by AgNPs and induction of apoptosis via caspase-dependent pathway^20^. In a previous investigation, *Bryophyllum pinnatum* mediated BP-AgNPs also displayed cytotoxicity against the same cell lines^49^. However, after an incubation period of 24 h, the respective IC_50_ values of BP-AgNPs against A431 and B16F10 were 59.5 and 96.6 µg/ml, almost double the IC_50_ values of RC@AgNPs. *Cucurbita maxima*, *Moringa oleifera,* and *Acorus calamus* mediated AgNPs were also found to inhibit A431 cell lines, but their respective IC_50_ values (82.39, 83.57, and 78.58 μg/ml) were higher than that of RC@AgNPs^50^. Similarly, *Indigofera hirsuta* fabricated AgNPs displayed lesser cytotoxicity (IC_50_ value: 80.9 μg/ml) against B16F10^51^.

Few reports are published on the antiproliferative activity of *R. cordifolia.* Cytotoxic activity of ZnO and CeO_2_ NPs from *R. cordifolia* leaf extract has been reported against MG-63, a human osteosarcoma cell line^52^. Cytotoxic activity of methanolic plant extracts of *R. cordifolia* is also reported against Hela and Hep-2 cell lines, with respective IC_50_ values of 23.12 and 11.92 µg/Ml^53^. *R. cordifolia* has shown antiproliferative activity against a wide range of cancer cells, such as human colon carcinoma (HT-29), human breast carcinoma (MCF-7), and human liver carcinoma (HepG2) cell lines, and human colon carcinoma (HT-29) ^54^. The probable mechanism for the antiproliferative activity might be inhibition of the DNA synthesis. *R. cordiofolia* can inhibit the incorporation of [3H] thymidine and c-fos gene expression, and the c-fos gene is responsible for the proliferation and differentiation of cells^55^.

Further, in a study done by Adwankar and Chitnis, they isolated a pure compound RC-18 from *R. cordifolia*, which was found to have antitumor activity against different types of in vivo solid tumor models (B16 melanoma)^53^. The cyclic hexapeptides of *R. cordifolia* were also reported for their anticancer activity by inhibiting the process of protein synthesis. It does so by binding to the 80 s subunit of the ribosome and thus inhibiting the binding of aminoacyl-tRNA and translocation of peptidyl-tRNA. Secondary metabolites found in *R. cordifolia* L., such as purpurin and munjistin have antitumor activity^20^. They may be contributing to the anticancer activity of *R. cordifolia* against different types of cancer^18–20^.

### DNA interaction capability

This is one of the most frequently used techniques to investigate the interactions between CT-DNA and NPs. This study relies on interaction to change or shift the maximum absorption of AgNPs. According to Topală et al., the hypochromic, hypsochromic (blue shift), and bathochromic (redshift) effects are caused by intercalative binding mode, whereas hyperchromism is caused by groove binding (minor and major), electrostatic interactions, and hydrogen bonding^56^. Chandraker et al. proposed that the stability of CT-DNA be tested at 15-min intervals before introducing NPs, and the absorption peak be studied for 1 hour^25^. Therefore, UV–Visible titration analyses were performed to examine the interaction between CR@AgNPs and CT-DNA.

**Figure 11:**
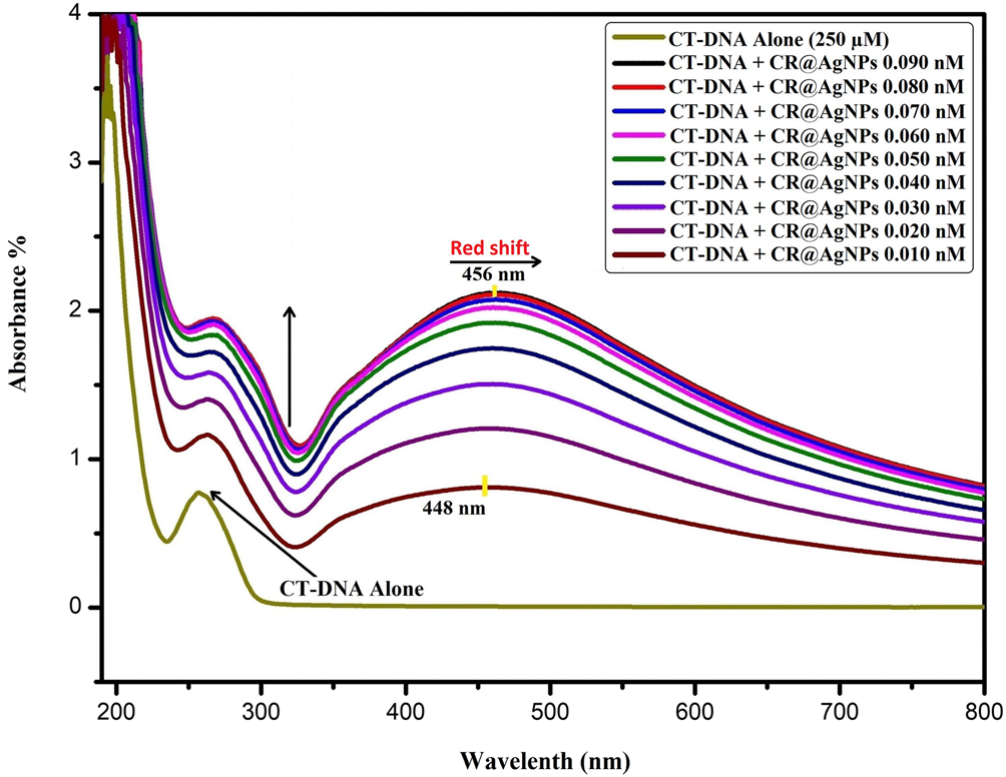
UV–Visible absorption spectra of interaction between CT-DNA (250 μM) and varying concentrations of RC@AgNPs.

To study the interactive effect of CR@AgNPs with CT-DNA, the varied concentration of CR@AgNPs (0.010 to 0.090 nM) was used. UV–visible spectra of CT-DNA at increasing concentrations of CR@AgNPs and constant CT-DNA 250 µM are shown in Fig. 11. The results indicate that when RC@AgNPs are introduced, the absorption at 253 nm increases without a detectable change in the value of λ_max_. According to Rahban et al. alteration in the secondary structure of DNA is due to hyperchromic effects^57^. The RC@AgNPs absorption spectra also showed a bathochromic effect from 453 to 463 nm. The hyperchromic effect in CT-DNA absorption spectra and the bathochromic effect in CR@AgNPs absorption spectra indicate a strong interaction between both. Only a few research on the interaction between NPs and DNA have been reported^58,59^. According to a prior study, the N7 atoms of guanine and adenine bases are likely contact sites in CT-DNA with NPs, while the N3 atoms of cytosine and thymine bases are involved in hydrogen bonding^25^.

### Antifungal activity of CR@AgNPs

**Figure 12:**
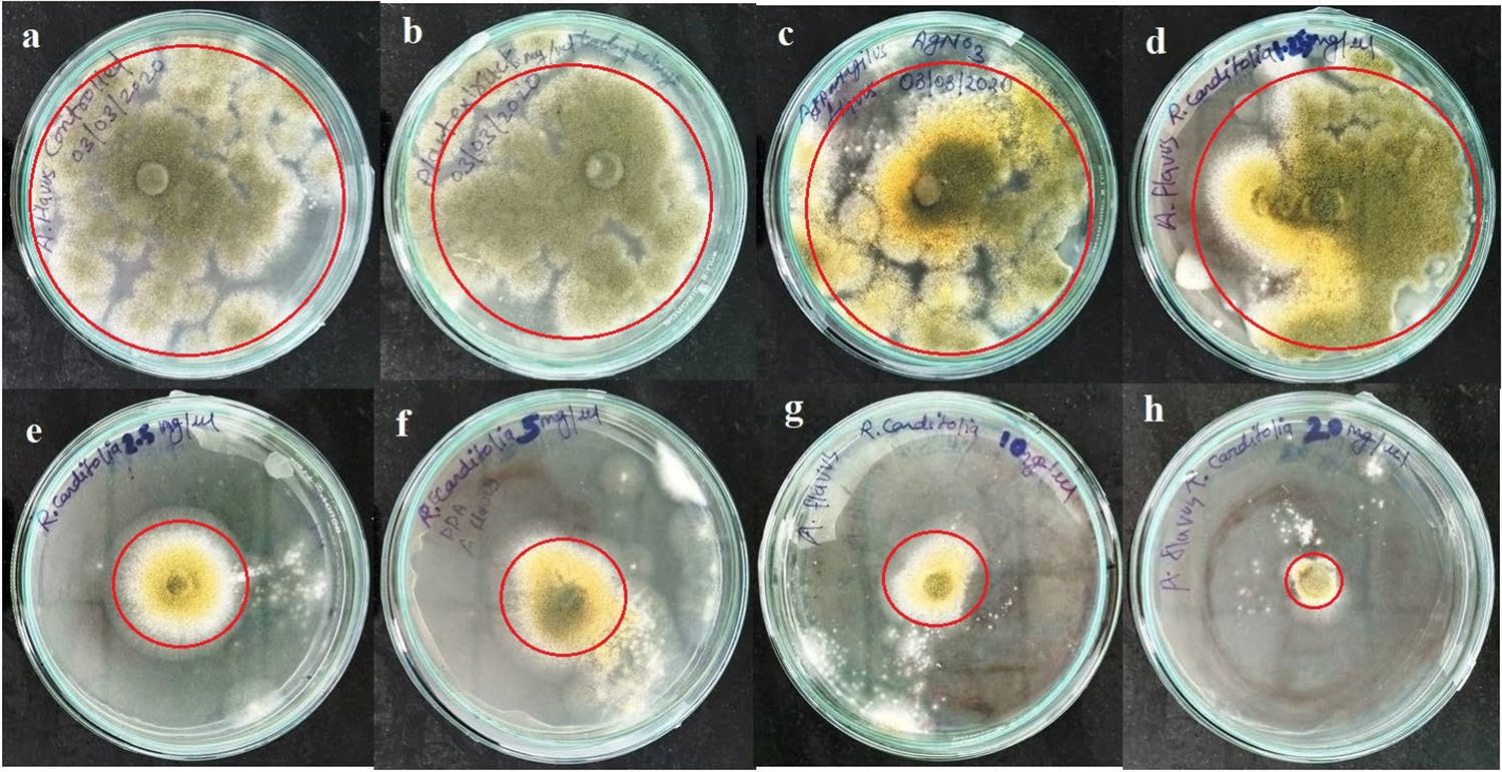
Antifungal activity of RC@AgNPs against: *A. flavus*. The rows represent (**a**) 0 (control), (**b**) RCLE, (**c**) AgNO_3_, and different concentrations of RC@AgNPs: (**d**) 0.0625, (**e**) 0.125, (**f**) 0.25, (**g**) 0.5, and (**h**) 1 mg/mL in PDA.

**Figure 13:**
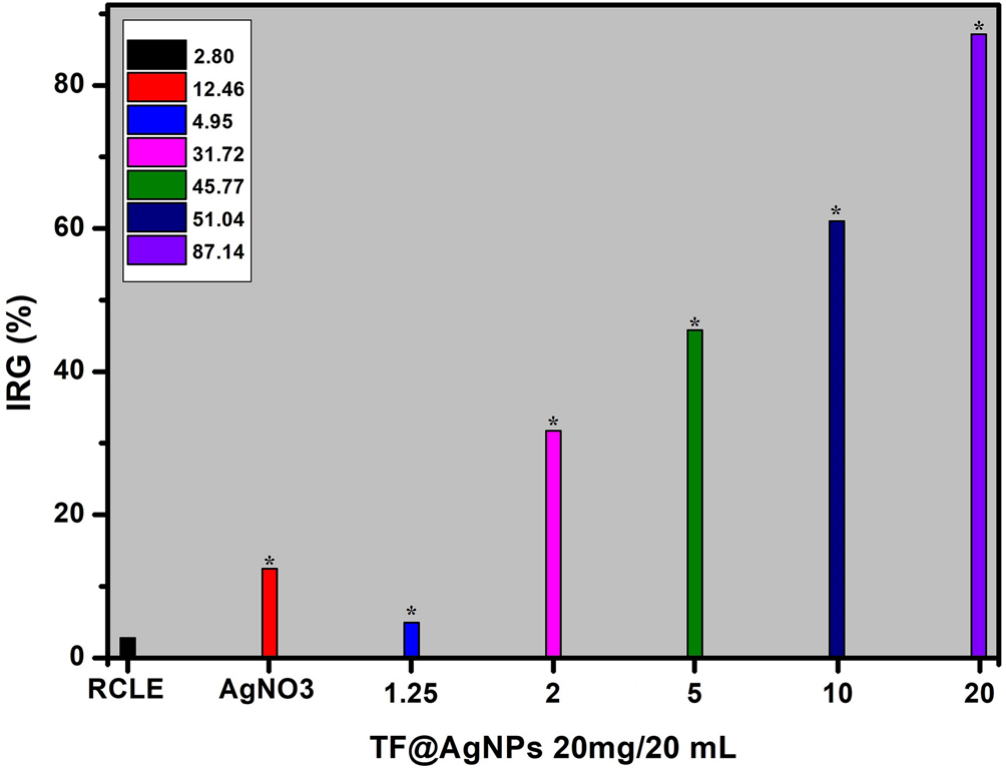
IRG (%) of RC@AgNPs against *A. flavus*, (*) indicates significant differences (P < 0.05) in comparison to their control.

*Aspergillus flavus* is a serious pathogen causing preharvest and postharvest infections of nuts, cereals, and legumes. Aflatoxins, produced by certain strains of *A. flavus* can lead to neutropenia, immunosuppression, acute hepatitis, and hepatocellular carcinona^60^. The antifungal activity of the phytofabricated RC@AgNPs was assessed by observing the radial growth of the mycelial colony in all the treatments. Figure 12 depicts the effect of RC@AgNP inhibition at various doses against *A. flavus* over a six-day incubation period. In our research, we found that the concentration of RC@AgNPs had a direct impact on *A. flavus* mycelia growth. When the concentrations of RC@AgNPs increased, the inhibition of radial growth (IRG) increased as well (Fig. 13). The color, shape, texture, form, and density of the fungal colony in treated plates differed from the control group. At a maximum dose of 1 mg/mL, the IRG of RC@AgNPs against *A. flavus* was 84.48%, whereas, at the minimum (0.0625 mg/mL), the IRG was recorded 37.17%. The IRG was found to be 21.59, 11.26, 42.71, 48.42, and 55.39% against RCLE, AgNO_3_, 0.125, 0.25, and 0.5 mg/mL concentration, respectively.

Very few reports are available on the fungitoxicity of AgNPs against aflatoxigenic *A. flavus*. *Kigellia pinnata* bark extract mediated AgNPs were used for the antifungal activity following the well-diffusion method^61^. *Corallocarpus epigaeus* mediated AgNPs showed only 46% inhibition of *A. flavus* at the same concentration we used^62^. Antifungal activities of *Ougeinia oojeinensis* and *Juniperus procera* mediated AgNPs were evaluated against other *Aspergillus* spp. (*A. niger* and *A. fumigatus*), respectively^63,64^. Jaffri and Ahmad followed a disc-diffusion assay to determine the fungitoxicity of prunosynthetic AgNPs against four *Aspergillus* spp^40^. Bocate et al. similarly studied the impact of AgNPs on various *Aspergillus* spp., but they used fungi-mediated AgNPs deploying *Fusarium oxysporum* and an antilipemic drug, simvastatin^60^.

### Antioxidant activity

**Figure 14:**
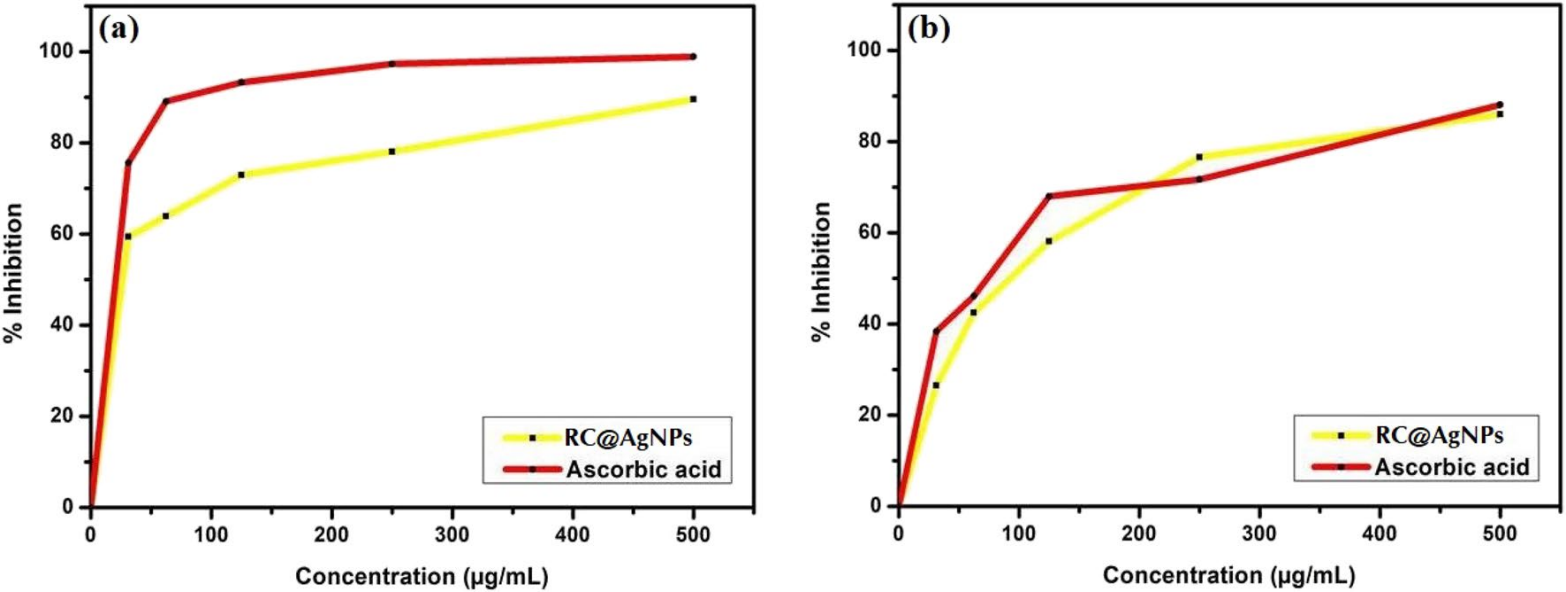
Antioxidant activity of RC@AgNPs, (**a**) DPPH assay, (**b**) ABTS assay.

*in-vitro* antioxidant activity of RC@AgNPs was assessed following DPPH and ABTS assays. Both the assays are comparatively quick and sensitive for analyzing the antioxidant activity of a particular substance compared to others^25^. The scavenging impact of RC@AgNPs on DPPH (Fig. 14a) and ABTS (Fig. 14b) free radicals was noticed in a dose-dependent manner. DPPH is a strong oxidant with an absorption wavelength range of 515–520 nm. between 515–520 nm. DPPH has a purple-blue color that changes into a bright yellow or colorless when it reacts with a substance having strong; stable free radical scavenging property^65^. The intensity of color transition is dependent on the total dose and nature of the sample. The DPPH radical inhibition of RC@AgNPs was 59.43 ± 0.296 to 89.58 ± 0.221% in the concentration range of 31.25 to 500 µg/mL. The same concentration of the standard reference (ascorbic acid) showed 75.64 ± 0.08 to 98.94 ± 0.03% inhibition (Fig. 14a). Similarly, at concentrations of 31.25 to 500 mg/mL of RC@AgNPs, ABTS free radical inhibition ranged from 26.54 ± 0.05 to 85.96 ± 0.10%. Ascorbic acid, on the other hand, was inhibited from 38.39 ± 0.05 to 88.09 ± 0.1% (Fig. 14b). AgNPs mediated by *Prosopis farcta* fruit extract, *Cucumis prophetarum* leaf extract, and blackcurrant pomace extract have similar DPPH free radical scavenging capabilities^66–68^. The capacity of the NPs to quench neutral and cationic radicals was examined, indicating that the RC@AgNPs may generate stable neutral radicals by DPPH and free cation radicals from ABTS. The antioxidant mechanism was distinct in both of the assays used. The DPPH test demonstrates AgNPs' ability to transfer electrons and neutralize reactive DPPH free radicals in the reaction medium^69^. The ABTS assay identifies cationic-free radical scavenging activities utilizing both el^-^ and H^+^ transport mechanisms^70^.

## Conclusions

*R. cordifolia*-mediated AgNPs were synthesized following a green synthesis and eco-friendly approach. The biogenic AgNPs are formed during the reduction of Ag^+^ ions by RCLE. The phytosynthesized RC@AgNPs were spherical with an average size of ~ 20.98 nm. Different biomedical activities (DNA-binding, antifungal, and antioxidant) of RC@AgNPs were explored, and it was found to have significant cytotoxic activity against skin cancer cell lines, A431 and B16F10. This is the first report on *R. Cordifolia* leaf-mediated biocompatible and bio-fabricated AgNPs. Based on the remarkable biological activities, RC@AgNPs is hereby recommended for its uses in biomedical applications with elaborated research. The study motivates further therapeutic research in the fields of cancer and antifungal agents.
